# Regulation and functions of alternative polyadenylation in fungi

**DOI:** 10.1080/21501203.2025.2486776

**Published:** 2025-04-04

**Authors:** Lei Zhang, Xiaoling Deng, Wenshuai Ma, Tianjiao Zhou, Chuan Xu

**Affiliations:** a Bio-X Institutes, Key Laboratory for the Genetics of Developmental and Neuropsychiatric Disorders, Ministry of Education, Shanghai Jiao Tong University, Shanghai, China; b Department of Otorhinolaryngology Head and Neck Surgery, Shanghai Sixth People’s Hospital Affiliated to Shanghai Jiao Tong University School of Medicine, Shanghai, China

**Keywords:** Alternative polyadenylation, fungi, gene regulation, functions, transcription

## Abstract

Alternative polyadenylation (APA) has been implicated in regulating transcriptome diversity and gene expression by significantly altering mRNA abundance, stability, localisation, and translation. Understanding the comprehensive landscape of APA can provide valuable insights into the complexity of gene regulation. In this review, we first outline the critical factors and mechanisms for the selection of PAS. Then, we summarise the experimental as well as the bioinformatic technologies for studying APA. In addition, we review and discuss current studies on fungi, aiming to highlight the role of APA in various biological processes, including growth and development, metabolism, responses to stress, and potential contribution towards virulence during host infection. Finally, we propose key questions along with perspectives for future research on APA in fungi. With more in-depth studies like comparative transcriptomic analyses, genome-wide poly(A) site mapping throughout the fungal kingdom, additional signatures and functions of APA will be uncovered, and its diverse roles will gradually come into focus.

## GRAPHICAL ABSTRACT

**Figure:**
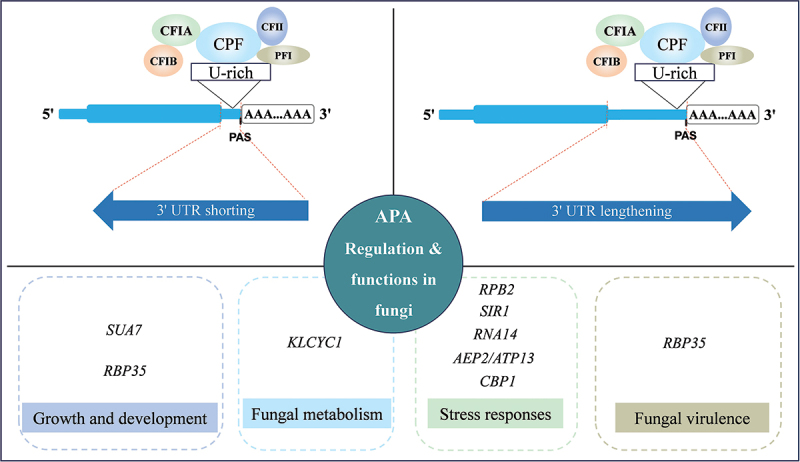

## 1. Introduction

Gene expression is a precisely controlled process by which the information encoded in DNA is converted into functional transcripts or proteins. Expression of protein-coding genes involves several key steps including transcription, 5’ capping, pre-RNA splicing, polyadenylation of 3’ end, mRNA nuclear export, and translation (Rodriguez-Molina et al. 2023). Polyadenylation, is a two-step process consisting of an endo-nucleolytic cleavage followed by the addition of a poly(A) tail at the 3’ end (Xu and Zhang 2018). The accurate regulation of polyadenylation is crucial for mRNA maturation, mRNA stability, mRNA export, and translation (Liu et al. 2022a). In eukaryotic organisms, ranging from fungi to humans, a multitude of genes exhibit the presence of multiple polyadenylation sites [poly(A) sites, PASs], termed as alternative polyadenylation (APA), culminating in the generation of diverse mRNA isoforms derived from a single gene (Guo and Lin 2023). Genome-wide analyses across eukaryotes have revealed that APA is more widespread than previously thought (Jan et al. 2011; Wu et al. 2011; Derti et al. 2012; Li et al. 2012). For example, in humans, approximately 70% of genes exhibit APA, with about 50% having three or more polyadenylation sites (Derti et al. 2012). Similarly, in Arabidopsis, 70% of genes use more than one poly(A) site (Wu et al. 2011).

**Figure 1.**
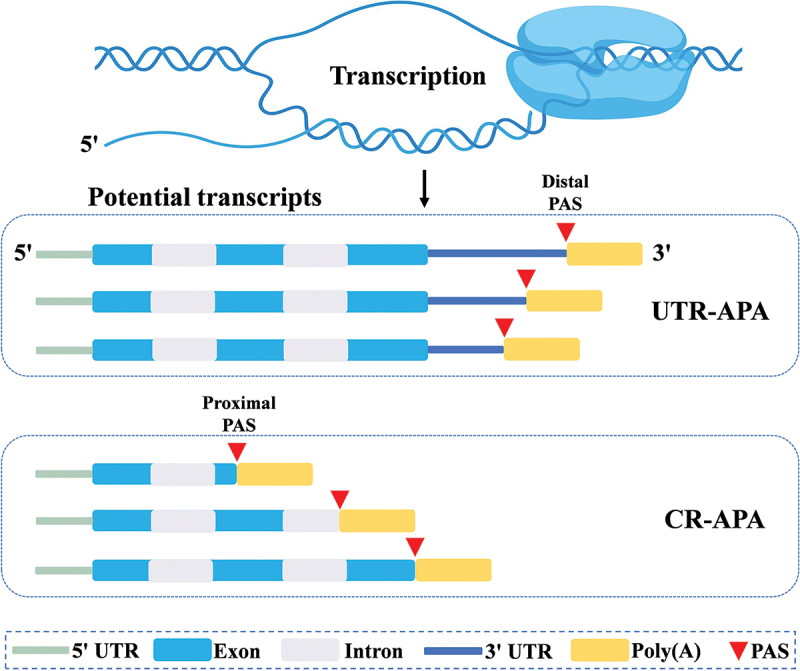
Types of alternative polyadenylation (APA) generated by alternative polyadenylation site (PAS). APA can be classified into untranslated region APA (UTR-APA) and coding region APA (CR-APA) when the PAS is located in the untranslated region (UTR) or coding region, respectively. Polyadenylation at the distal PAS results in longer transcripts, while polyadenylation at the proximal PAS generates shorter transcripts.

APA has recently been recognised to play an important role in the regulation of gene expression. According to the location of poly(A) sites along genes, APA can be classified into two major categories (Figure 1) (Chen et al. 2017). When alternative PASs are situated within internal introns or exons, this leads to the production of different mRNA isoforms which can be translated into distinct protein isoforms (Guo and Lin 2023). This type of APA is termed coding region-APA (CR-APA). Conversely, when the PAS are located within the 3’ untranslated region (3’ UTR), it results in transcripts with varying 3’ UTR lengths but encodes the same protein. This type of APA is referred to as UTR-APA (Chen et al. 2017). While CR-APA can affect gene expression qualitatively by producing distinct protein isoforms, UTR-APA has the potential to affect expression quantitatively (Tian et al. 2005; Tian and Manley 2017). Thus, APA is a widespread regulatory mechanism that significantly enriches the transcriptome and promotes diversities.

The roles of APA in the regulation of gene expression underscore its importance in many biological contexts, including cell proliferation, tissue differentiation, and adaptation to stress (Muzafar et al. 2021; Kowalski et al. 2024). For instance, studies have demonstrated that during early embryonic development, translation efficiency is closely linked to poly(A) tail length, with mRNAs possessing longer poly(A) tails generally being translated more efficiently than those with shorter tails (Xiang and Bartel 2021). In non-dividing yeasts under nutrient-restricted conditions where cells reach a quiescent state, there is a preference for the expression of longer 3’ UTR (Liu et al. 2017). Moreover, malfunctions in APA regulation can lead to various human pathologies, including but not only tumorigenesis and neurodegenerative diseases (Yeh and Yong 2016; Yeganeh Markid et al. 2024). A global reduction in the length of the 3’ UTR has been documented in various types of cancer (Yeh and Yong 2016). Interestingly, the progression of Alzheimer’s disease is associated with the elongation of 3’ UTRs within the central nervous system (Yeganeh Markid et al. 2024). Thus, understanding the comprehensive landscape of APA could lead to novel therapeutic strategies to combat diseases associated with its dysregulation.

APA has been investigated across a spectrum of eukaryotes, ranging from fungi to humans, including classic experimental models like budding yeast *Saccharomyces cerevisiae* (*S. cerevisiae*) and fission yeast *Schizosaccharomyces pombe* (*S. pombe*) (Liu et al. 2017), plant pathogens such as *Magnaporthe oryzae* (*M. oryzae*) (Franceschetti et al. 2011) and *Fusarium graminearum* (*F. graminearum*) (Lu et al. 2022), vertebrate oocytes (Li et al. 2012), and various cancer cells (Mayr and Bartel 2009; Zingone et al. 2021). Interestingly, pathogenic fungi during the process of infecting host also displays huge variations in PAS numbers and APA densities, suggesting a potential role for APA in modulating fungal virulence (Rodriguez-Romero et al. 2019). In addition, the advances in sequencing technology, bioinformatics as well as the availability of several sequenced genomes have allowed a closer look at 3’ end processing of mRNA in fungi. Due to the importance of model fungi in understanding eukaryotic gene regulation and hazard posed by pathogenic fungi, a comprehensive understanding of the roles of APA in fungi is essential.

In this review, we provide a summary of currently available studies in fungi that aim to highlight the role of APA in various biological processes. First, we outline the critical factors and mechanisms for the selection of PAS. Then we provide an overview of the most commonly used methods for studying APA, including both experimental approaches and bioinformatics tools. Finally, we summarise the functions of APA involved in fungal biology including growth and development, metabolism, responses to stresses, and its possible contribution towards virulence during host infection.

## 2. Pre-mRNA 3’ end processing in fungi

**Figure 2.**
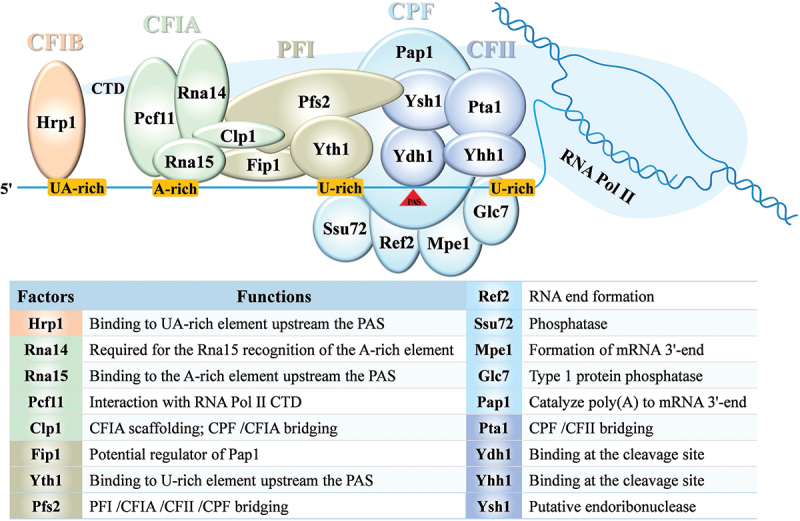
Schematic representation of the known machinery for yeast 3’ end processing. The RNA *cis*-elements are highlighted in chrome yellow, including the ua-rich element, the A-rich element, and the U-rich element upstream of the polyadenylation site (PAS), and the downstream U-rich element. The key *trans*-acting factors including cleavage factor IB (CFIB), cleavage factor IA (CFIA), polyadenylation factor I (PFI), cleavage and polyadenylation factor (CPF), and cleavage factor II (CFII) are colour-coded, with different subunits of the same complex marked in the same colour. Their respective functions are listed in the table below, with the full name of each subcomplex provided in the main text.

APA isoforms from the same gene may either encode different protein variants or exhibit unique functions due to the distinct 3’ UTRs. Since longer 3‘ UTRs often contain more binding sites for microRNAs and RNA-binding proteins than shorter 3’ UTRs, APA isoforms may exhibit differences in stability, translation efficiency, and/or intracellular localisation (Tian and Manley 2013). Due to the importance of APA in gene expression, understanding how PAS is recognised and how PAS selection is regulated are crucial. An increasing number of APA-regulatory factors in fungi have been discovered and studied, including the *cis*-elements and the *trans*-factors, such as 3’ end-processing factors, Poly(A) binding proteins, transcription factors, and splicing factors (Figure 2). These factors can either broadly regulate APA or specifically affect the APA of certain genes (Stroup and Ji 2023).

### 2.1. Cis-elements

The cleavage and polyadenylation (CPA) sites at the 3’ end are defined by *cis*-elements located in the 3‘ UTR. These sites are determined through the recognition of specific PAS within nascent transcripts by a multiprotein core complex and numerous associated factors (Tian and Graber 2012). Several key *cis*-regulatory elements have been experimentally identified, and to describe them clearly in fungi, drawing analogies to mammals may be necessary. In mammals, 3’ end processing is driven mainly by three *cis*-elements. The canonical PAS hexamer (AAUAAA and its variants) is the most important for site selection. It is usually found 15–30 nucleotides upstream of the cleavage site. Meanwhile, a core U/GU-rich downstream element (DSE) is typically located about 14–70 nucleotides downstream. The cleavage site is located between the PAS and DSE, and the cleavage happens just upstream of an adenosine residue (Wang et al. 2018). Additionally, the upstream UGUA element, which lies upstream of the PAS, plays a key role in recruiting both core and auxiliary polyadenylation factors to the cleavage site (Danckwardt et al. 2008). Together, these *cis*-elements establish the functional PAS, which is recognised by *trans*-acting factors to facilitate the coupled CPA reaction (Gruber and Zavolan 2019; Mitschka and Mayr 2022). In fungi, despite the apparent differences in the sequence and position of *cis*-elements, the 3‘ end processing machinery shares many similarities with mammals (Millevoi and Vagner 2010). Using yeast as a model organism, previous global analyses of yeast UTRs have identified four essential elements required for positioning the 3’ end processing machinery (Figure 2) (Graber et al. 1999; van Helden et al. 2000). These *cis*-elements, including UA repeats (UA-rich), consist of an A-rich region and U-rich segments, and are recognised by various processing factors. Located at a variable distance upstream of the PAS, the UA repeats specifically interact with Hrp1, which is the only subunit of yeast CFIB (Kessler et al. 1997). After the UA repeats, there is an A-rich sequence, with a preference for the motif AAUAAN, which, although similar to the AAUAAA hexamer found in mammals, is more degenerate in yeast. A similar phenomenon of increased degeneracy is also observed in plants, making yeast poly(A) signals more similar to those of plants than mammals, as both yeast and plants lack downstream elements (Graber et al. 1999). Interestingly, this A-rich element does not interact with CFII (the yeast homolog of CPSF) but instead associates with CFIA, a homolog of the mammalian CstF complex. In addition, two U-rich elements flanking the PAS are essential for efficient 3’ end processing (Dichtl and Keller 2001).

### 2.2. Trans*-acting factors*

A variety of *trans*-acting factors are needed to carry out CPA reaction of the 3’ end in yeast, including CFIA (Cleavage Factor IA), CFIB (Cleavage Factor IB), CFII (Cleavage Factor II), PFI (Polyadenylation Factor I), and CPF (Cleavage and Polyadenylation Factor), all of which participate in the CPA reaction as multi-subunit complexes (Zhao et al. 1999; Mayr and Bartel 2009). CFIA consists of Rna14, Rna15, Clp1, and Pcf11, interacts with the A-rich element and facilitates the polyadenylation process; CFIB consists of Hrp1 factor, which binds to UA repeats and promotes the positioning of PAS; CFII consists of Pta1 (Pre-Trna Accumulation 1), Yhh1 (Cft1, Cleavage Factor Two 1), Ydh1 (Cft2, Cleavage Factor Two 2), and Ysh1 (Yeast 73 kDa Homolog 1), binds to the downstream U-rich elements and promotes efficient 3’ end cleavage and polyadenylation in yeast. PFI consists of Fip1 (Factor Interacting with Poly(A) polymerase 1), Yth1 (Yeast 30 kDa Homolog 1), and Pfs2 (Polyadenylation Factor Subunit 2) (Ghazy et al. 2009). CPF consists of Ref2 (RNA End Formation 2), Ssu72 (Suppressor of SUa7, gene 2), Mpe1 (Mutant PCF11 Extragenic suppressor), Glc7 (GLyCogen), and Pap1 (Poly(A) Polymerase) (Lingner et al. 1991; Vo et al. 2001; Darmon and Lutz 2012).

Using yeast as a model, the details of interactions between *cis*-elements and *trans*-factors are summarised in Figure 2. Although multiple factors and mechanisms have been discovered in fungal species, our understanding of fungal APA mechanisms remain limited compared to mammals. With the advancement of sequencing technologies and analytical approaches, it is anticipated that new mechanisms will continue to be discovered. Exploring new APA mechanisms, identifying auxiliary factors involved in PAS selection, and elucidating the molecular pathways that govern their activity and expression will continually clarify our understanding of the complexities of PAS selection in fungi.

## 3. Technologies and methods for studying APA

Although the importance of APA has long been recognised, comprehensive and detailed studies on APA only began to emerge with the widespread adoption of high-throughput sequencing technologies (Chen et al. 2017; Shah et al. 2021). In the past decade, the significantly reduced cost and broad applicability of bulk RNA sequencing (RNA-seq) have made it a standard approach in molecular biology research (Moon et al. 2024). Recent studies have utilised RNA-seq to investigate APA, enabling the identification of poly(A) tail locations. Many labs have recently focused on developing computational tools for the identification and quantification of PAS from routine RNA-seq, including TAPAS (Arefeen et al. 2018), DaPars (Xia et al. 2014), QAPA (Ha et al. 2018), APAlyzer (Wang and Tian 2020), and APAtrap (Ye et al. 2018), and others (see details in Table 1). Moreover, all of the aforementioned tools are equipped with the capability to predict PASs and identify differential APA usage. They can process millions of poly(A)-containing reads and detect thousands of putative APA cleavage sites. For the most accurate PAS identification, TAPAS is recommended. For optimal PAS quantification, QAPA is preferred. DaPars2 is the tool of choice for unannotated genes or de novo PAS discovery, while APATrap is the most suitable option for studies involving plant species.

**Table 1.** Computational tools for predicting or identifying APA site and/or dynamic APA events.

Tools	Full-name	Advantages	Disadvantages	Data Types	Website	Reference
TAPAS	Tool for Alternative Polyadenylation site Analysis	Good PAS recovery rate	High computational resource requirements	RNA-seq data	https://github.com/arefeen/TAPAS.	Arefeen et al. (2018)
DaPars2	Dynamic analyses of Alternative PolyAdenylation from RNA-Seq	Effective for de novo identification of PAS	Requires paired-end sequencing to improve accuracy	RNA-seq data	https://github.com/ZhengXia/DaPars.	Xia et al. (2014)
Roar	Roar: detecting alternative polyadenylation with standard mRNA sequencing libraries	Effective for comparing short and long 3’ UTRs	Limited ability to identify novel PAS	RNA-seq data	https://github.com/vodkatad/roar.	Grassi et al. (2016)
PAQR2	PolyAdenylation site usage Quantification from RNA sequencing data	Improved accuracy and high reproducibility	High computational resource demand	RNA-seq data	https://github.com/zavolanlab/PAQR2.	Gruber et al. (2018)
GETUTR	Global Estimation of The 3’ UTR landscape based on RNA-seq	Effectively reconstructs 3’ UTR structure	Lower sensitivity in PAS detection	RNA-seq data	http://big.hanyang.ac.kr/GETUTR.	Kim et al. (2015)
APAtrap	Alternative PolyAdenylation Trap	Particularly effective for plant species	Performs poorly with mammalian species	RNA-seq data	https://apatrap.sourceforge.io.	Ye et al. (2018)
QAPA	Quantification of APA	Easy to use with high computational efficiency	Less effective for unannotated genes	RNA-seq data	https://github.com/morrislab/qapa.	Ha et al. (2018)
APAlyzer	Alternative PolyAdenylation analyzer	High-quality PAS annotation relies on the PolyA_DB database	Limited ability to identify novel PAS	RNA-seq data	https://github.com/RJWANGbioinfo/APAlyzer/.	Wang and Tian (2020)
polyA-miner	polyA-miner	Detection of novel APA sites	Requires high sequencing depth	mRNA 3’-seq data	https://github.com/YalamanchiliLab/PolyA-miner.	Yalamanchili et al. (2020)
QuantifyPoly(A)	QuantifyPoly(A)	Better resolution of microheterogeneity	Requires more processing power and memory	mRNA 3’-seq data	https://sourceforge.net/projects/quantifypoly-a/.	Ye et al. (2021)
IntMAP	Integrative Model for Alternative Polyadenylation	High accuracy	High computational resource demand	RNA-seq and mRNA 3’-seq data	https://github.com/kuanglab/IntMAP.	Chang et al. (2018)
APA-scan	Alternative PolyAdenylation scan	High accuracy	Greater computational complexity	RNA-seq and mRNA 3’-seq data	https://github.com/compbiolabucf/APA-Scan.	Fahmi et al. (2022)
scDaPars	Single-Cell Dynamic analyses of Alternative PolyAdenylation from RNA-Seq	Single-cell resolution	Greater computational complexity	Single-cell RNA-Seq	https://github.com/YiPeng-Gao/scDaPars/.	Gao et al. (2021)
scAPAtrap	Single-Cell Alternative PolyAdenylation Trap	Single-cell resolution	Greater computational complexity	Single-cell RNA-Seq	https://github.com/BMILAB/scAPAtrap/.	Wu et al. (2021)
SCINPAS	Single Cell Identification of Novel Poly(A) Sites	Single-cell resolution	Greater computational complexity	Single-cell RNA-Seq	https://github.com/zavolanlab/SCINPAS/.	Moon et al. (2023)

RNA-seq has provided a powerful tool for transcript analysis, yet it still has certain limitations in precisely mapping APA sites across the entire genome, where the precision of de novo PAS identification remains relatively low. Therefore, improved targeted mRNA 3’-seq methods have been developed to enhance the sequencing accuracy of mRNA 3‘ ends, such as 3’ READS (Hoque et al. 2013), IVT-SAPAS (Fu et al. 2015), Quantseq (Moll et al. 2014), 3P-seq (Spies et al. 2013), PAS-seq (Shepard et al. 2011), and polyA-seq (Derti et al. 2012). A summary of these methods, along with key characteristics, is provided in Table 2. For researchers prioritising the most accurate PAS identification, 3‘ READS is the preferred method despite its experimental complexity. When RNA availability is limited, IVT-SAPAS serves as a suitable alternative due to its compatibility with low-input RNA samples. In contrast, for large-scale studies, such as large cohort studies, QuantSeq is advantageous for its efficiency in handling high-throughput samples. These 3’ end targeted approaches, which primarily detect 3’ ends of transcripts, have been employed to efficiently investigate the global effects of variations on APA. Meanwhile, several improved tools have been developed for 3‘ end targeted techniques, such as polyA-miner (Yalamanchili et al. 2020), QuantifyPoly(A) (Ye et al. 2021), IntMAP (Chang et al. 2018), and APA-Scan (Fahmi et al. 2022). Each tool employs distinct algorithms and offers unique strengths, yet their overall methodologies share common principles (see Table 1 for details). These typically include steps such as data pre-processing, PAS identification, PAS clustering, and the analysis of differential PAS usage. Notably, both IntMAP and APA-Scan integrate RNA-seq with targeted mRNA 3’-seq data, allowing for high-accuracy and quantitative profiling of APA events. For example, in the analysis of BT549 breast cancer cells, the combination of 3’-end-seq and RNA-seq data suggests that APA-Scan can effectively reduce false-positive events (Chang et al. 2018; Fahmi et al. 2022).

**Table 2.** mRNA 3’-seq library preparation methods.

Methods	Full-name	Characteristics	Sequencing strategy	Reference
3’ READS	3’ Region Extraction And Deep Sequencing	Use the CU_5_T_45_, bypass the internal priming events.	Fewer A/T residues remain, allowing for normal sequencing.	Hoque et al. (2013)
3P-seq	Poly(A)-Position Profiling	Avoid internal priming by splint ligation.	Fewer A/T residues remain, allowing for normal sequencing.	Jan et al. (2011)
PAT-seq	Poly(A)-test RNA-sequencing	Preserving strand information and minimise internal priming.	Fewer A/T residues remain, allowing for normal sequencing.	Harrison et al. (2015)
IVT-SAPAS	In Vitro Transcription Sequencing APA Sites	Combining *in* *vitro* transcription so that the RNA input can be less.	Introduce non-A nucleotides in the poly(A) and sequencing normally.	Fu et al. (2015)
SAPAS	Sequencing APA Sites	Template switching to generate the double-strand cDNA library with adapters and primers.	Introduce non-A nucleotides in the poly(A) and sequencing normally.	Fu et al. (2011)
Quantseq	Quantseq	Commercialized standard oligo(dT)-based priming using a random primer for second-strand cDNA synthesis.	Use the custom sequencing primer.	Moll et al. (2014)
2P-seq	Poly(A)-tail-Primed sequencing	Reverse transcription of RNA fragments with an oligo(dT) primer, followed by cDNA circularisation.	Use the custom sequencing primer.	Spies et al. (2013)
PAS-seq	Poly(A) Site sequencing	Use the SMART reverse transcription system which is based in template switching.	Use the custom sequencing primer.	Shepard et al. (2011)
MAPS	Multiplex Analysis of PolyA-linked Sequences	Biotinylated oligo-dT initially used to prime cDNA synthesis and sequencing.	Sequencing starts from the opposite end of the T stretch.	Fox-Walsh et al. (2011)
A-seq	A-seq	Ligation of 5’ adaptors to RNA fragments, followed by reverse transcription with a split oligo(dT) primer that contains the 3’ adaptor.	Sequencing starts from the opposite end of the T stretch.	Martin et al. (2012)
A-seq2	A-seq2	Use oligo(dT) primer which contains a dU within Ts for the selection of poly(A)+ RNA fragments and reverse transcription.	Use USER enzyme to remove T-stretch and add four random nucleotides before sequencing.	Gruber et al. (2014)
3’ T-fill	3’ T-fill	Fragmented RNA is reverse transcribed with an oligo(dT) primer coupled to adapter and biotin.	The poly(A) stretch is filled in with dTTPs before sequencing.	Wilkening et al. (2013)
PAC-seq	Poly(A) Click sequencing	Reverse transcription using an oligo(dT) primer in combination with spiked-in azido-nucleotides (AzVTPs), which induce termination of the reverse transcription process.	Sequencing from the opposite end of the T stretch.	Routh et al. (2017)
PolyA-seq	PolyAdenylation sequencing	RNase H treatment followed by second strand synthesis.	Use the custom sequencing primer.	Derti et al. (2012)

The rapid advancement of 3’ tag-based single-cell RNA sequencing (scRNA-seq) technologies like CEL-seq and 10× Genomics has enabled researchers to increasingly utilise scRNA-seq data to investigate cell-type- and development-stage-specific APA events. This shift has catalysed the development of computational approaches aimed at identifying APA sites and analysing APA dynamics at single-cell resolution. Consequently, several innovative computational tools, such as scDaPars (Gao et al. 2021), scAPAtrap (Wu et al. 2021), and SCINPAS (Moon et al. 2023), can quantify and assess APA usage at both single-cell and single-gene resolution. Moreover, another promising method for profiling APA is Direct RNA Sequencing (DRS), developed by Helicos Bioscience and Oxford Nanopore Technology (Ozsolak et al. 2010; Polenkowski et al. 2023). DRS avoids the known biases and artefacts introduced during RNA measurements by reverse transcription, PCR, or other sample manipulation steps (Cocquet et al. 2006; Wu et al. 2008; Mamanova et al. 2010). These emerging sequencing methods offer unparalleled spatial and temporal insights into the expression of APA isoforms, marking a significant advancement in APA research.

## 4. Functions of APA in fungi

### 4.1. Widespread of APA in fungal species

Recent studies have revealed that APA is widespread in model fungal species (Ozsolak et al. 2010; Liu et al. 2017). For instance, in two key model organisms, *S. cerevisiae* and *S. pombe*, approximately 84.7% and 82.4% of protein-coding genes exhibit APA, respectively, underscoring the prevalence of APA in these two central models. These APA sites are predominantly located in the 3’ UTRs, generating isoforms with variable 3’ UTR lengths. Specifically, 78% of protein-coding genes in *S. cerevisiae* and 71% in *S. pombe* were found to produce multiple 3’ UTR isoforms. On average, each protein-coding gene in *S. cerevisiae* harbors approximately 2.6 distinct APA sites within its 3’ UTR, while those in *S. pombe* contain around 2.5 such sites. These results further highlight the importance of these models in understanding the extensive role of APA in regulating gene expression (Liu et al. 2017).

APA was also observed in plant pathogenic fungus. For instance, Rodriguez-Romero et al. used a 3’ T-fill method to identify 14,593 PAS in filamentous fungi *M. oryzae*, finding that 52% (4,283) of its genes were alternatively polyadenylated (Franceschetti et al. 2011). In a similar study of *F. graminearum*, they identified 364,513 unique PAS and observed that 64.8% (11,133) of genes exhibited APA. On average, each gene in *F. graminearum* had 9.2 PAS, with 6,123 PAS identified in perithecia and 5,530 in hyphae (Lu et al. 2022). These findings emphasise the widespread occurrence of APA in plant pathogenic fungi in PAS usage. These results suggest that APA is pervasive in fungi as in plants and mammals. With APA being a widespread phenomenon, it has been found to play a crucial role in various aspects of fungal biological processes, and an increasing number of genes are being identified as associated with it, some of these known genes are listed in Table 3.

**Table 3.** Genes with APA related to fungal biological processes.

Functions	Genes	Species	Reference
Growth and development	*SUA7*	*Saccharomyces cerevisiae*	Hoopes et al. (2000)
	*RBP35*	*Magnaporthe oryzae*	Franceschetti et al. (2011)
Metabolism	*KLCYC1*	*Kluyveromyces lactis*	Seoane et al. (2005)
Stress	*CBP1*	*Saccharomyces cerevisiae*	Sparks et al. (1997)
	*AEP2/ATP13*	*Saccharomyces cerevisiae*	Sparks and Dieckmann (1998)
	*RNA14*	*Saccharomyces cerevisiae*	Sparks and Dieckmann (1998)
	*SIR1*	*Saccharomyces cerevisiae*	Sparks and Dieckmann (1998)
	*RPB2*	*Saccharomyces cerevisiae*	Yu and Volkert (2013)
Virulence	*RBP35*	*Magnaporthe oryzae*	Franceschetti et al. (2011)

### 4.2. APA in growth and development

The fungal developmental process is regulated by numerous RNA processing events, which encompass various single and combinatory APA patterns (Franceschetti et al. 2011; Liu et al. 2017). In *M. oryzae*, the rice blast fungus, it has been reported that more than half of the multi-exon genes produce alternatively polyadenylated mRNA isoforms. These APA genes are notably enriched in several critical biological pathways, including mitotic cell cycle regulation, protein targeting and translocation, budding, cell polarity, and filament formation (Franceschetti et al. 2011). These pathways are crucial for regulating key biological processes, such as mycelium development, sporulation, and responses to environmental signals (Rodriguez-Romero et al. 2019). Notably, APA in the 5’ UTRs often serves a regulatory function by generating upstream open reading frames (uORFs) (Rodriguez-Romero et al. 2015). uORFs can modulate gene expression by inhibiting the translation of downstream coding sequences. For example, the *RBP35* gene in *M. oryzae* produces two polyadenylated transcripts within its 5’ UTR intron, and these transcripts are upregulated in response to growth inhibition caused by carbon starvation. One of these transcripts, encoding uORF1, was found to be essential for restoring fungal growth in the presence of rapamycin, underscoring the significant role of APA in regulating fungal growth and proliferation (Rodriguez-Romero et al. 2015).

Another study has highlighted the significant role of APA in regulating ageing and dormancy in *F. graminearum*. During these processes there is a significant increase in the use of distal PAS, resulting in the production of longer 3’ UTR isoforms. This shift in PAS usage was particularly evident in dormant conidia and hyphal tissues over a 24 to 48 h period. The genes showing increased distal PAS usage in these states are frequently associated with pathways involved in senescence and aging, similar to those observed in mammals (Deschenes and Chabot 2017; Chen et al. 2018; Angarola and Anczukow 2021; Lu et al. 2022). These findings suggest that APA-driven 3’ UTR lengthening is crucial for regulating fungal development and physiological transitions, such as dormancy and ageing, thus affecting the fungus’s growth and survival.

More intriguingly, a recent study on the plant wilt-causing pathogen *Verticillium dahliae* revealed that the transcription factor VdCf2 regulates its growth, pathogenicity, and the expression of a putative gene cluster involved in secondary metabolism. Additionally, VdCf2 has been shown to interact with the gene *VDAG_07278*, which is known to generate multiple isoforms via APA. This has sparked a series of associations and speculations regarding the impact of APA regulation on fungal growth. For example, different mRNA isoforms of *VDAG_07278* may result in variations in the translated products, thereby altering their interaction patterns with VdCf2. Such dynamic changes could influence key signalling pathways associated with fungal growth, morphogenesis, and environmental adaptation. Although there is currently no direct evidence linking the APA phenomenon of *VDAG_07278* to the growth and development of this fungus, this characteristic provides an important clue for exploring the potential regulatory functions of APA in fungal growth (Jin et al. 2017; Liu et al. 2022b).

### 4.3. APA in fungal metabolism

The critical roles of APA in regulating metabolic processes in fungi have been investigated in various yeast species (Seoane et al. 2005; Liu et al. 2017; Lester 2023). In *S. cerevisiae*, the most enriched biological process (BP) terms of genes with APA-patterns include “organic cyclic compound biosynthesis process”, “organic cyclic compound metabolic process”, and “response to drug”, indicating the significant role of APA in yeast metabolism (Liu et al. 2017). Another yeast, *Kluyveromyces lactis*, is widely used in biotechnological applications, such as the production of aromatic compounds, secretion of heterologous proteins, and removal of lactose from industrially discarded milk whey. The Respiro-fermentative metabolism of this yeast has been extensively studied. Notably, cytochrome c encoded by *KlCYC1* is an essential component for growth under respiratory conditions. The long 3’ UTR of *KlCYC1* is crucial for maintaining normal Respiro-fermentative metabolism (Freire-Picos et al. 2001; Seoane et al. 2005).

In the plant pathogenic fungus *M. oryzae*, APA also plays a crucial role in regulating the expression of genes involved in key metabolic pathways, particularly nitrogen metabolism. The Target of Rapamycin (TOR) pathway, which is a central regulator of cellular growth in response to environment by modulating primarily protein synthesis, autophagy and metabolism (Zoncu et al. 2011; Shimobayashi and Hall 2014). Recent research shows that the absence of the gene-specific polyadenylation factor RBP35 results in dysregulation of 3’ UTRs in TOR signalling pathway genes, which further leads to the significant changes in nitrogen metabolism and protein secretion. These findings highlight the important role of APA in regulating fungal metabolism. However, the lack of clear RBP35 orthologues in yeast suggests that RBP35 is a novel auxiliary protein of the polyadenylation machinery in filamentous fungi, thereby regulating the nitrogen metabolism pathway (Franceschetti et al. 2011; Marroquin-Guzman and Wilson 2015).

### 4.4. APA events mediated by environment stress in fungi

All cells inherently monitor nutrient availability and adapt to fluctuations in nutrient levels as a core regulatory function (Fafournoux et al. 2000; Hatzoglou et al. 2004; Lopez et al. 2007). Fungi, in particular, exhibit remarkable plasticity under challenging environmental conditions, which is a crucial factor in their success as pathogens and in their ability to adapt to new environments (Muzafar et al. 2021). APA appears to play a key role in these adaptive processes, helping fungi adjust to various stress conditions. Stresses come in many forms, such as altered nutrient availability, temperature fluctuation, unstable genome, and oxygen depletion. At the cellular level, a range of proteins are synthesised in response to these stressors, directing the cell’s survival and recovery (de Nadal et al. 2011; von Roretz et al. 2011). A common feature of stress responses is the suppression of general gene expression mechanisms, coupled with the selective activation of genes essential for cell survival (de Nadal et al. 2011). In this context, fungal cells have been shown to regulate APA dynamically in response to diverse environmental stimuli (Mayer and Dieckmann 1991; Sparks and Dieckmann 1998; Hoopes et al. 2000; Liu et al. 2017; Rodriguez-Romero et al. 2019).

The evidence for the regulation of APA induced by various stressors is well established. A comparison of APA patterns in two yeast species, *S. cerevisiae* and *S. pombe*, revealed that changes in PAS selection within 3’ UTRs are influenced by nutrient availability. In both yeast models, the transition from nitrogen-deprived to nitrogen-rich conditions predominantly led to 3‘ UTR shortening events (Liu et al. 2017). Specifically, the yeast *CBP1* gene is transcribed into two distinct isoforms through APA, with the shorter transcript being specifically enriched under respiratory growth conditions. Similarly, APA of several other genes in yeast including *SUA7*, *AEP2/ATP13*, *RNA14*, and *SIR1*, is modulated by different carbon sources (Sparks and Dieckmann 1998; Hoopes et al. 2000). Interestingly, a study in the filamentous ascomycete *M. oryzae* revealed that there is a strong preference for distal PAS usage during carbon starvation, regulating up to 553 alternatively polyadenylated genes (Rodriguez-Romero et al. 2019). In addition, UV damage has been reported to regulate the APA event in *S. cerevisiae*, causing a preference for longer 3’ UTRs in the *RPB2* gene (Yu and Volkert 2013). Similarly, a genome-wide analysis of APA in *S. cerevisiae* showed that more genes prefer shorter 3’ UTRs under heat shock conditions, while under oxidative stress, there is an opposite preference for longer 3’ UTRs (Waern and Snyder 2013).

Taken together, APA primarily influences 3’ UTRs during stress by modulating transcript composition through the inclusion or exclusion of regulatory elements, thereby refining gene expression to support cell survival.

### 4.5. APA in regulation of fungal virulence

Understanding microbial virulence is essential for the development of novel diagnostic methods and innovative therapeutic strategies. With the advent of modern technologies, systems biology-based analyses of host-pathogen interactions have become increasingly significant. Recent studies have highlighted that APA plays a pivotal role in regulating the pathogenicity and virulence of fungal pathogens (Franceschetti et al. 2011; Rodriguez-Romero et al. 2019). For instance, in *M. oryzae*, RBP35 mediated APA plays a crucial role during host plant infection. This factor regulates both the expression and distal PAS selection of genes involved in virulence and signalling (Franceschetti et al. 2011). The importance of RBP35 in virulence was further demonstrated by screening *M. oryzae* mutants impaired in root infection; specifically, *Δrbp35* mutants exhibit a preference for proximal PAS selection and failed to induce spreading necrotic lesions on rice leaves, suggesting that the loss of RBP35 almost completely eliminates the virulence of *M. oryzae* (Fernandez et al. 2014).

Additional genes in *M. oryzae*, such as *MST7*, *14-3-3A*, and *14-3-3B*, also exhibit significant changes in APA during the infection process. Among these, *14-3-3B* shows the most pronounced alterations, and further investigation revealed that its longer 3’ UTR is crucial for the full expression of disease symptoms, suggesting a heightened level of virulence (Rodriguez-Romero et al. 2019). This indicates that APA-driven variations in 3’ UTR length may allow *M. oryzae* to finely tune gene expression in response to the dynamic host environment. Moreover, targeting the APA machinery or specific APA events in virulence-associated genes could provide novel strategies for controlling fungal infections, highlighting the importance of further research into the broader role of APA in fungal biology and host-pathogen interactions.

## 5. Conclusion and future perspective

### 5.1. Functional similarity of APA events in fungi compared to other eukaryotes

Global analyses of APA in eukaryotes have highlighted its significant role in gene regulation across species. Notably, orthologous human and mouse genes exhibit a high similarity in the number of mapped 3’ ends (Tian et al. 2005; Ara et al. 2006), suggesting that APA sites have been actively selected during evolution.

The functional diversity generated by APA is crucial for various cellular processes. For instance, genes expressed across multiple tissues exhibited different APA preferences, with those in the retina, placenta, blood, and ovary more likely to use proximal PAS, whereas genes in the bone marrow, uterus, brain, and nervous system showed increased usage of distal PAS (Di Giammartino et al. 2011). Proliferating cells also favour proximal PAS, while quiescent or differentiated cells show increased use of distal sites, and 3’ UTRs generally lengthen during embryonic development (Li et al. 2012). Similarly, in fungi, different tissues and developmental stages display distinct patterns of PAS usage (Lu et al. 2022). Moreover, eukaryotic cells are exposed to various stressors under both physiological and pathological conditions, including oxidative stress, heat shock, and endoplasmic reticulum stress, all of which have been associated with ageing and diseases such as cancer, cardiovascular disorders, and neurological conditions. In response to these stressors, specific genes crucial for maintaining cell survival and homoeostasis undergo transcriptional activation (Zheng et al. 2018). Studies show that stress impacts APA in both plants (de Lorenzo et al. 2017) and mammalian cells (Hollerer et al. 2016), suggesting APA is a key adaptive mechanism for coping with environmental stress. Additionally, various cellular conditions, including cell senescence, neuronal activation, and viral infections, can alter global APA profiles (Lianoglou et al. 2013; Zheng et al. 2018). Likewise, stress-induced alterations in PAS usage are quite common in fungi (Thiebaut et al. 2008; Graber et al. 2013; Waern and Snyder 2013; Rodriguez-Romero et al. 2019). All the similarities above suggest the functions of APA events are conserved across different eukaryotes in evolution.

Notably, while *S. cerevisiae* and *S. pombe* do not possess homologs of the mammalian Cleavage Factor I complex (CFIm), a putative CFIm25-related homolog has been identified in the filamentous fungus *Aspergillus oryzae* as well as the plant pathogens *Ustilago maydis* and *M. oryzae*, indicating some conservation of this factor during evolutionary process (Munsterkotter and Steinberg 2007; Franceschetti et al. 2011). Conversely, certain regulatory factors remain specific to yeast, such as Ref2, which directly binds RNA and is essential for efficient processing at weak PAS in yeast (Russnak et al. 1995). Another factor, RBP35, which lacks clear orthologs in yeast, plants, and animals, has been identified as a novel auxiliary protein of the polyadenylation machinery in filamentous fungi. It has been demonstrated to be functionally equivalent to the metazoan CFI 68 kDa, further suggesting the existence of 3’ end processing mechanisms unique to the fungal kingdom (Franceschetti et al. 2011).

### 5.2. Unanswered questions in APA

The fungal kingdom encompasses an estimated 5 million species, including unicellular yeasts, filamentous fungi, large-structured mushrooms, and pathogens that infect both plants and animals, exhibiting remarkable phenotypic diversity and complexity (O’Brien et al. 2005). Although APA has been extensively studied in model yeasts, research on filamentous fungi remains limited. While some studies have investigated APA in *M. oryzae* and *F. graminearum*, the broader field remains underexplored. Given its significant impact on human health and daily life, further research on APA in filamentous fungi is crucial. Since APA plays a role throughout various developmental stages, it likely contributes to phenotypic variation, environmental adaptability, and pathogenic potential, underscoring the need for deeper exploration in this field (Franceschetti et al. 2011; Liu et al. 2017; Rodriguez-Romero et al. 2019).

Due to the tight link between the CPA reaction and transcription termination, it has generally been assumed that most regulated APA events occur in a co-transcriptional manner. However, a recent study in human cells indicates that while CPA at distal PAS primarily occurs co-transcriptionally, CPA at alternative proximal PAS can take place either during transcription or as a post-transcriptional event (Tang et al. 2022). This new insight prompts further exploration into related biological questions, such as whether this mechanism is unique to humans or a universal process shared across species, and whether it also occurs in other organisms, including fungi. More critically, the biological functions and physiological significance of post-transcriptional polyadenylation remain to be fully understood. One potential hypothesis suggests that additional processing at the proximal PAS serves as a regulatory mechanism, facilitating a rapid response to specific stimuli without requiring de novo transcription, particularly when sequential polyadenylation is induced. Nonetheless, this hypothesis warrants further investigation to uncover its underlying mechanisms and implications (Hao et al. 2023).

Although APA has been shown to significantly impact the pathogenicity of fungi, most studies have focused on plant pathogenic fungi, with a notable scarcity of research on APA in animal pathogenic fungi, such as *Candida albicans* (*C. albicans*). To better understand the role of APA in human fungal pathogens, key questions remain. For example, how does APA regulate virulence genes in pathogens like *C. albicans* and *Cryptococcus neoformans*? Additionally, does APA contribute to antifungal resistance? Answering these questions could provide valuable insights into the pathogenic mechanisms of fungi and potentially lead to the development of more targeted therapeutic strategies. Specifically, understanding how APA regulates virulence and resistance could reveal new drug targets, offering novel approaches for treating fungal infections in humans.

### 5.3. A demand of optimal approaches for studying APA

In the past decade, the rapid development of high-throughput sequencing technologies has provided us with the opportunity to study APA in depth. However, the diverse sequencing methods for APA and the variety of data processing tools have led to inconsistencies in PAS identification, quantification, and usage within the same studies, which can be highly problematic. It is highly challenging to choose an optimal bioinformatic approach for APA analysis. Currently, no software is available that can seamlessly handle different sequencing datasets, significantly limiting our research and understanding of the precise mechanisms and functions of APA. In addition, although significant advances in fungal biology have been made through various genomic and sequencing approaches, these methods alone are insufficient to address many biological questions. The recent breakthrough in genome editing technologies, such as CRISPR, allows for precise manipulation of specific regions and PASs, holding great promise for furthering our understanding of APA in fungal biology.

In conclusion, as APA is increasingly recognised for its widespread occurrence and potential role in shaping phenotypic complexity, further investigation into gene-specific isoform regulation is crucial. Such studies could shed light on unresolved questions in cell biology, offer deeper insights into gene expression mechanisms, and pave the way for gene-targeted therapeutic strategies. Moreover, deciphering APA expression patterns will enhance our understanding of gene function and may uncover their broader implications for human health.
